# A Low-Cost Layered Double Hydroxide (LDH) Based Amperometric Sensor for the Detection of Isoproturon in Water Using Carbon Paste Modified Electrode

**DOI:** 10.1155/2020/8068137

**Published:** 2020-08-20

**Authors:** Herve Leclerc Tcheumi, Aude Peggy Kameni Wendji, Ignas Kenfack Tonle, Emmanuel Ngameni

**Affiliations:** ^1^Laboratoire de Chimie Analytique, Département de Chimie Inorganique, Faculté de Sciences, Université de Yaoundé I, BP 812 Yaoundé, Yaoundé, Cameroon; ^2^Laboratoire de Chimie de l'Environnement, Département des Sciences Environnementales, Ecole Nationale Supérieure Polytechnique de Maroua, Université de Maroua, BP 46 Maroua, Maroua, Cameroon; ^3^Laboratoire de Chimie Minérale, Département de Chimie, Faculté des Sciences, Université de Dschang, BP 67 Dschang, Dschang, Cameroon

## Abstract

In this work, a Layered Double Hydroxide (NiAl-LDH) was obtained by coprecipitation method and used to elaborate an electrochemical sensor for the determination of isoproturon, which is a hazardous pollutant, widely used in agriculture, and its residue is distributed into aqueous environment through run-off and leaching from the soil. Various physicochemical techniques such as FT-IR spectroscopy, X-ray diffraction, and thermal analysis were used to characterize this material. The anionic exchange capacity of NiAl-LDH on carbon paste modified electrode was investigated toward [Fe(CN)_6_]^3-^ using cyclic voltammetry. Used as electrode modifier of carbon paste electrode for isoproturon detection, a remarkable increase in isoproturon signal on modified carbon paste electrode by LDH was observed. The peak current obtained after 3 min of preconcentration in 25 *μ*M ISO on NiAl-LDH/CPE was 2.6 times higher than that exhibited by the same analyte on the unmodified CPE, thereby opening the way to the development of a sensitive method for the detection of ISO. Other parameters that can affect the stripping response (preconcentration time, pH of detection medium, and LDH loading within the paste) were investigated to optimize the proposed sensor. After optimization, a linear calibration curve was obtained in the concentration range from 2 × 10^−8^ to 1.8 × 10^−7^ M, leading to a detection limit of 1 × 10^−9^ M (S/N = 3). The relative standard deviation for 5 identical measurements was 2.7%. The interfering effect of some compounds and ions was examined on the stripping response of ISO. The applicability of the method was verified by the determination of ISO in spiked water sample.

## 1. Introduction

Isoproturon [3-(4 isopropylphenyl)-1,1-dimethylurea, referred to as ISO hereafter] is a phenylurea herbicide widely used for pre- and postemergence control of annual grasses and broad-leaved weeds in spring and water cereals [1]. Progressive increase in the production and application of ISO for plant protection induces the problem of water quality due to the long time taken by this class of compounds to degrade [2]. The intensive use of phenylurea herbicides as ISO in agriculture results in a high risk of these herbicides entering the food chain by means of contaminated water [3]. Its residues can cause severe health effects in both human and animals upon its absorption [4], due to its chronic toxicity, carcinogenicity, and genotoxicity [5]. Therefore, sensitive determination of ISO is highly important. Up to now, several techniques have been reported for phenylurea herbicides determination; they include ultraviolet spectroscopy [5], capillary electrophoresis [3,6], and mainly gas or liquid chromatography [7,8]. Nevertheless, these methods are not easy to carry out because they often necessitate long analysis times and require several preprocessing steps and expensive equipment. Additionally, these methods are not sensitive. Therefore, the development of simple, low cost, and sensitive methodologies for monitoring of pesticides remains a daily concern. Following these lines, electrochemical analysis has significant advantages such as speediness and less expensive instrumentation. However, the unmodified electrodes suffer from drawbacks such as high overpotential and electrodes surfaces fouling issues. As such, finding suitable electrode modified for determination of organic pollutants is an interesting research. In that context, Sundari and Manisankar [9] elaborated a modified electrode by coating multiwalled carbon nanotube film on a glassy electrode for electrochemical determination of ISO; Noyrod et al. [10] reported a simultaneous determination of ISO and carbendazim by single drop analysis using a graphene-based electrochemical sensor. Promising research results have been obtained with electrodes modified with Layered Double Hydroxides (LDHs) for electroanalysis of organic and inorganic pollutants as have also been explored [11–14], because of their ability to accumulate various chemical species in their interlayer region. In fact, LDHs are the class of host-guest type layered material consisting of positively charged metal hydroxide layers acting with free hydrated anions located in the interlayer space. A general formula for the most widely studied LDHs is [M^II^_1−x_ M^III^_x_ (OH)_2_ ]^x+^.[(A^n−^) _x/n_. mH_2_O] where M^II^ can be Mg^2+^, Mn^2+^, Ni^2+^, etc., while M^III^ may be Al^3+^, Mn^3+^, Cr^3+^, etc. A^n−^ represents an intercalable anion, and x is the molar ration. Recently, considerable interest has been diverted to the study of the adsorption of environmental contaminant by LDH materials [15]; however, most of them were focused on the adsorption of anion pollutants. As a result, it is necessary to design and synthesize adsorbents based on LDH materials for adsorption of neutral pollutants.

Several approaches have been used to synthetize LDH; these include coprecipitation hydrothermal method, urea hydrolysis, and electrodeposition [16]. Nevertheless, coprecipitation is a technique that is widely used to make batches materials due to its relative simplicity and ease of use of the resulting material for adsorption of pollutant in batch adsorption mode [17]. The development of electrochemical sensors for ISO at trace level has attracted an increasing interest in the last decade. Different methods have been proposed to modify electrode surface with LDH film [12, 13]; the most common one consists of depositing a fixed amount of a colloidal solution of the LDH, previously synthesized in bulk by the coprecipitation method [18], onto the support. This method suffers a drawback of poor adhesion of the film to the support material, thus lacking the reproducibility of the results obtained. To overcome of this weakness of film modifier electrode, Carbon Pate Electrode (CPE) presents interesting alternative. In fact, carbon paste electrode possesses many advantages which include low background current, rapid and confortable renewal of the active surface of the electrode, and easy fabrication and modification with a wide range of compounds. Investigations dealing with the use of LDH as modifier of CPE for sensing of pollutant are not widespread. To overcome these, numerous attempts have been made. For example, Fuerte et al. [11] investigated the electrochemical oxidation of 4-chlorophenol using CPE modified with ZnAl-LDH and Isa et al. [14] used multiwalled carbon nanotube modified with ZnAl-LDH to prepare chemically modified carbon for Hg^2+^ determination.

This study designed and developed an electrochemical sensor for ISO detection using CPE modifier electrode with NiAl-LDH. The ISO retention by NiAl-LDH was through H-bonding between the hydroxyl group NiAl-LDH and amide group of ISO. This type of interaction can contribute to the enhancement of the sensitivity of ISO during the detection step. NiAl-LDH synthetized was fully characterized (FT-IR, XRD, and TGA) prior to their application as electrode material and used for the development of a sensitive sensor dedicated to the electrochemical determination of ISO in water samples.

## 2. Experimental Methods

### 2.1. Chemicals and Reagents

All chemicals and reagents used in this work were of analytical grade and used as received. ISO was purchased from Supelco, France, and 10^−2^ M stock solutions were prepared in methanol. K_3_Fe(CN)_6_ (>99%, Prolabo) was reagent grade and used as received. An acetate buffer solution was used as supporting electrolyte and was prepared by mixing 0.1 mol L^−1^ CH_3_COONa and CH_3_COOH (Riedel-de-Haen). The pH of solution was adjusted using molar NaOH and HNO_3_ solutions prepared from analytical reagents purchased, respectively, from BDH and Prolabo. All the aqueous solutions were prepared using deionized water.

### 2.2. Synthesis of NiAl-LDH and Their Characterization

The synthesis of NiAl-LDH by a conventional coprecipitation method was adapted to the method previously reported [19]. In practice, an aqueous solution of nickel nitrate and aluminum nitrate in the molar ratio 3/1 was prepared by dissolving, respectively, 0.0045 mol and 0.015 mol of these compounds in 100 ml of deionized water, the ph being maintained constant at 10.1 ± 0.5 by the addition of a sodium hydroxide solution (2 m). The synthesis was carried out using boiling water and under nitrogen atmosphere in order to minimize the contamination with atmospheric CO_2_. The resulting suspension was then stirred for 16 h and filtered and the solid obtained was collected, washed, and dried in an oven at 70°c for 24 h.

NiAl-LDH was subsequently characterized by X-ray powder diffraction (XRD), Fourier Transform Infrared (FT-IR), and thermal analysis.

XRD patterns were recorded at room temperature using a classical powder diffractometer (X'PERT PRO, Philips) equipped with a Cu anode (quartz monochromator, k*α*_1_ radiation, *λ* = 1.54056 Å).

Diffuse reflectance infrared spectra were recorded between 4000 and 500 cm^−1^, using a FT-IR Perkin Elmer 2000 spectrometer equipped with a DTGS detector. The sample was analyzed at room temperature using KBr pellets. The diffuse reflectance R_s_ of the sample and R_r_ of KBr used as nonabsorbing reference powder were measured in the same conditions. The spectrum resolution was 4 cm^−1^ and the accumulation time was 5 min.

Thermal Gravimetric Analysis (TGA) was performed on a SDT simultaneous DSC-TGA instrument under N_2_ flow (100 mL.min^−1^). Approximately 20 mg of the NiAl-LDH was placed on the thermobalance of analyzer, which was purged with helium gas.

### 2.3. Working Electrode Preparation, Electrochemical Equipment and Procedures

The preparation of CPE modified by LDH (namely, NiAl-LDH/CPE) was similar to the procedures previously described [20]. Briefly, CPE was obtained by intimately mixing a carbon powder from Alfa (particle size < 325 mesh), a pasting liquid (silicone oil, Prolabo), and LDH particles (60-30-10% w/w). The mixture was homogenized in a mortar for 40 min and placed in a cylindrical Teflon tube (6 mm internal diameter), equipped with a stainless steel screw and piston acting as the electrical contact. The active surface of the electrode was then polished on a sheet of clean paper.

The electrochemical measurements were performed on a *μ*-Autolab potentiostat controlled by the GPES software. Cyclic voltammetry was used to examine the electrochemical behavior of ISO, while square wave voltammetry was employed to optimize the sensitivity of sensor. Electrochemical procedure for sensing ISO was as follow: the modified electrode was dipped in a beaker containing 25 *μ*M of aqueous solution of ISO (50 mL) and maintained under mild stirring for 5 min for accumulation at open circuit. After this step, the electrode was removed, rinsed with distilled water, and then transferred to the electrochemical cell containing the detection solution. Prior to the SWV experiment, the detection medium was deaerated with nitrogen for 3 min. The regeneration of sensor was made by transferring the modified electrode (after detection) to a blank acetate solution under mild stirring. The trace amounts of ISO were totally desorbed after 1 min, shown by a flat voltammogram recorded after transferring the electrode from the detection medium.

## 3. Results and Discussions

### 3.1. Physicochemical Characterization of the NiAl-LDH

**Figure 1:**
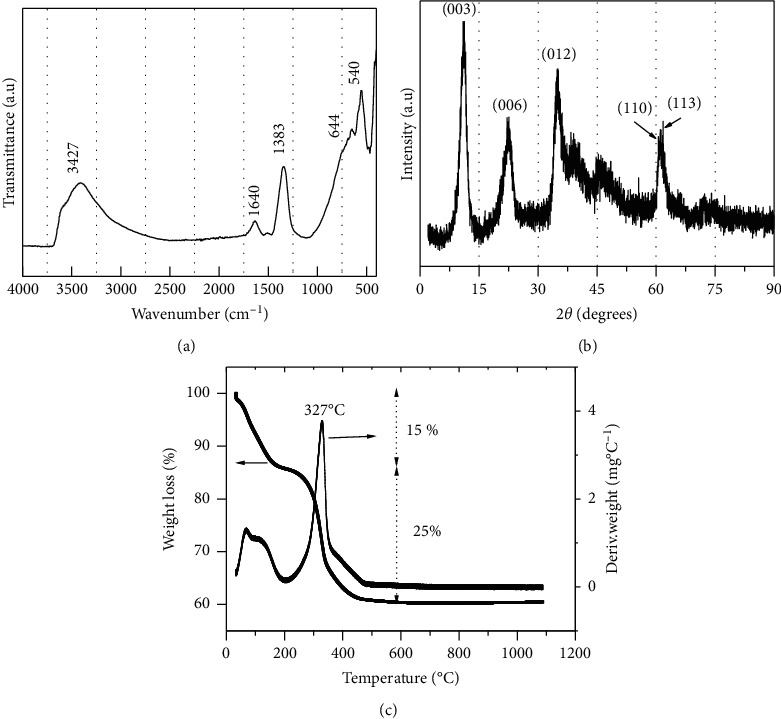
(a) Infrared spectra (4000-500 cm^−1^ region); (b) X-ray diffraction; (c) TG and DTG patterns of NiAl-LDH.

Figure 1(a) shows the FT-IR spectra of NiAl-LDH. A large absorption band which is centered at 3427 cm^−1^ corresponds to the stretching vibrations mode of hydrogen bonded physisorbed and intercalated water molecules [21]. Similarly, the band close to 1640 cm^−1^ corresponds to the O–H bending vibration of the interlayer water [22]. The intense band at 1383 cm^−1^ is due to the Ѵ_3_ vibration mode of nitrate ion in D_3h_ symmetry present in the interlayer space [23]. The band characteristic to metal-oxygen bond stretching appears at 644 cm^−1^ and 540 cm^−1^ is caused by various lattice vibrations associated with metal hydroxide sheets [24].

The XDR pattern of NiAl-LDH is presented in Figure 1(b). The peaks at 11.03° and 22.7° 2*θ* are assigned to the 003 and 006 reflections, respectively [25]. The first peak corresponds to a d_003_ value of 8.2 Ǻ. Also noted is the presence of well-defined reflections—012, 110, and 113—that are frequently used to confirm the good crystallinity of LDH [26].

The result of thermal analysis of material is presented in Figure 1(c). NiAl-LDH displayed a progressive mass loss of 15% in the temperature range between 20°C and 190°C, which is attributed to the loss of water molecules adsorbed on the external surface of NiAl-LDH or in the interlayer surfaces. From 250°C to 450°C, significant mass losses (25%) in two stages were observed, corresponding to the dehydroxylation and the loss of nitrate ions [27, 28]. The first event is fast and characterized by a well-defined DTG with a peak at 327°C, while the second is much slower with a broad and poorly defined peak centered at 400°C.

### 3.2. Electrochemical Characterization of NiAl-LDH

**Figure 2:**
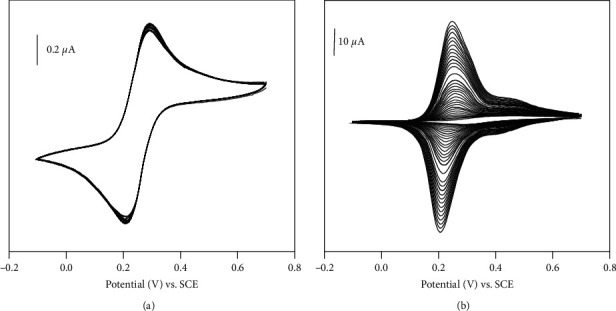
Multisweep cyclic voltammograms recorded at 50 mV s^−1^ in NaCl 0.1 M + 10^−4^ M of [Fe(CN)_6_]^3-^: (a) CPE; (b) NiAl-LDH/CPE. Scan rate 50 mV/s.

In order to explore the possible use of the NiAl-LDH material as modifier of CPE, some preliminary experiments were performed using [Fe(CN)_6_]^3−^ ions as electroactive probe. The reactivity of a material at a given modified electrode strongly depends on the properties of that material. Thus to get precise information about this material ion exchange voltammetry was used. This was done by examining the electrochemical behavior of [Fe(CN)_6_]^3−^ ions at modified and unmodified carbon paste electrode, by recording a series of cyclic voltammograms.  Figure 2 displays multisweep cyclic voltammograms recorded at bare CPE (Figure 2(a)) and NiAl-LDH/CPE (Figure 2(b)). At bare CPE, well-defined diffusion controlled redox behaviors with [Fe(CN)_6_]^3-^ with a constant steady state were recorded upon repetitive scanning. The cyclic voltammogram recorded at bare CPE were centered at E° = ½(E_pc_ + E_pa_) = 0.245 V with peak to peak separation values of ΔEp = (E_pa−_E_pc_) = 0.09 V at scan 50 mV/s and the magnitude of i_pa_/i_pc_ is equal to 1.09; this value suggests a reversible electrochemical system as a result of monoelectronic transformation involving [Fe(CN)_6_]^3−^and [Fe(CN)_6_]^4−^. When the electrode was modified by NiAl-LDH, the multisweep cyclic voltammetry performed on the same probe solution led to a different behavior as shown in Figure 2(b). During the first scan, a poor signal was observed due to the effect caused by nonconductive NiAl-LDH material. Since this material is an excellent anionic exchanger, gradual increase in the intensities of the signals with the number of scan is observed. The peak current reaches its maximum value after 40 cycles and the current was 49 times higher than that recorded for the unmodified CPE. The peak to peak separation recorded at NiAl-LDH/CPE was 0.045 V. This value was lower for about 45 mV compared with that obtained at bare CPE, suggesting that the diffusion of the probe is easy at modified electrode. This result is a proof that NiAl-LDH accumulates [Fe(CN)_6_]^3−^ ions by anion exchange mechanism [29, 30].

The active area of electrodes was studied using Randles–Sevcik equation. The cyclic voltammetry at varying sweep rates was recorded using K_3_[Fe(CN)_6_] solution 10^−3^ M in KCl solution 0.1 M. From the slope of the plot of Ip vs. *ν*1/2 (Figure S1), the A value was calculated using the following equation:

(1) $$\begin{array}{c} i_{p}=\left(2.69.10^{5}\right)D_{0}n^{\left(3/2\right)}C_{0}A. \end{array}$$

Here, for [Fe(CN)_6_]^3−^ = 10^−3^M, D_o_ = 7.6.10^−6^ cm^2^/s, and *n* = 1, the calculated effective surface areas were found to be 0.047 cm^2^ and 0.055 cm^2^ for bare CPE and with NiAl-LDH/CPE, respectively.

### 3.3. Electrochemical Behavior of Isoproturon on NiAl-LDH/CPE

**Figure 3:**
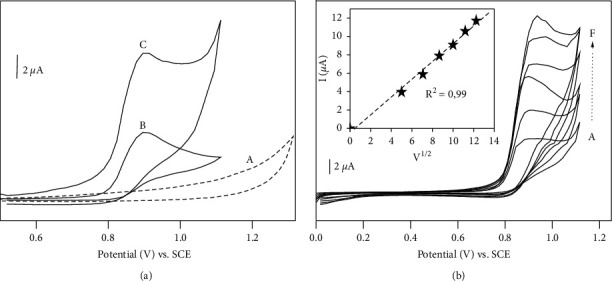
(a) Cyclic voltammograms recorded on (A) NiAl-LDH/CPE in 0.1 M acetate buffer (pH 4.5); (B) on CPE in 50 *μ*M ISO + 0.1 M acetate buffer (pH 4.5); (C) on NiAl-LDH/CPE in 50 *μ*M ISO + 0.1 M acetate buffer (pH 4.5); potential scan rate 50 mV s^−1^. (b) Influence of scan rate (v) on peak current of 50 *μ*M of ISO on the NiAl-LDH/CPE (curves a-f, *v* = 25, 50, 75, 100, 125, and 150 mv.s^−1^, respectively). Inset: plot of the anodic peak current (Ip_a_) versus v^1/2^.

Cyclic voltammogram of 50 *μ*M of ISO in acetate buffer at CPE and NiAl-LDH/CPE was recorded as shown in Figure 3(a). The blank cyclic voltammogram obtained at NiAl-LDH/CPE in acetate buffer is included (dotted line). In both cases, the electrochemical response shows one main anodic peak centered on 0.9 V. Such electrochemical behavior of ISO was similar to previous work described in the literature with phenylurea compound such as diuron and fenuron [3, 31]. The equation of this transformation was illustrated by (2) [32]. From this cyclic voltammogram, it is obvious that oxidation peak current is greater for NiAl-LDH/CPE than EPC. The peak current on NiAl-LDH/CPE was 3.25 times higher than that recorder on CPE, indicating an effective preconcentration ability of ISO by NiAl-LDH/CPE. The increase in peak current may be due to adsorptive ability of NiAl-LDH through hydrogen bonding between hydroxyl group of NiAl-LDH and amide group of ISO.

(2) 

In order to yield more insights in the electrochemical behavior of ISO, further experiments were performed; these include the studying of the effect of increasing the potential scan rate. From Figure 3(b), it appears that the peak current increases with the potential scan rate. A plot of the anodic peak current versus V^1/2^ exhibits a linear dependence as indicated by the graph in Figure 3(b), indicating that electrochemical oxidation takes place via adsorption with mass transfer [32]. From the above cyclic voltammetry studied, it appears that NiAl-LDH used as electrode modifier can be suitable for building an electrochemical sensor for ISO.

**Figure 4:**
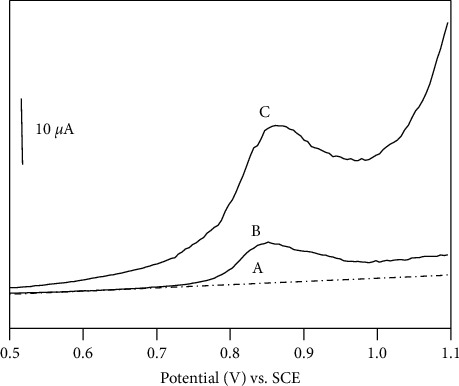
SWV response of (a) 0.1 M acetate buffer on CPE; (b) ISO 25 *μ*M after 3 min accumulation and detection in 0.1 M acetate buffer on CPE; (c) ISO 25 *μ*M after 3 min accumulation and detection in 0.1 M acetate buffer on NiAl-LDH/CPE. Other experimental conditions: electrolysis potential: 0.8 (V) pulse amplitude: 50 mV, frequency 125 Hz.

Figure 4 presents the SWV curves of acetate buffer solution at a CPE (Figure 4(a)) and 25 *μ*M ISO in acetate solution at a CPE (Figure 4(b)) or NiAl-LDH/CPE (Figure 4(c)). A well-defined but rather low peak was obtained in the potential range of 0.5 to 1.1 V with CPE (Figure 4(b)) pointing out the weak sorption capacity of the carbon towards ISO. When the CPE was modified by NiAl-LDH, its sensitivity was significantly improved (Figure 4(c)). The peak current measured was found to be equal to 13 *μ*A; this value is more than 2.6 times higher than that recorded at CPE (5 *μ*A).

**Figure 5:**
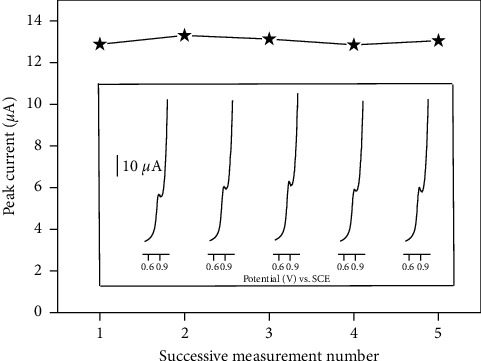
Typical successive peaks recorded using 25 *μ*M of ISO after 3 min accumulation and detection in 0.1 M acetate buffer on NiAl-LDH/CP; inset SWV response recorded in condition the same as in Figure 3.

Some important physicochemical parameters involved in the stripping process were examined in order to optimize the sensitivity of the modified electrode, in view of its possible use as ISO sensor. Prior to this study, the stability and reproducibility of the signal of ISO on the NiAl-LDH/CPE were evaluated before its application to the voltammetric detection of ISO. Thus, a series of five successive SWV experiments of the same electrode were performed in 25 *μ*M of ISO solution (Figure 5), and a coefficient of variation of 2% was noticed, indicating that the modified electrode has good reproducibility. The stability of the electrode was also investigated by measuring the electrode response with 25 *μ*M of ISO every day. Between the measurements, the electrode was stored at 4°C in a refrigerator. The current response decreases to 5% after 5 days.

### 3.4. Optimization of Experimental Parameters for ISO Detection

#### 3.4.1. Influence of the Accumulation Time

**Figure 6:**
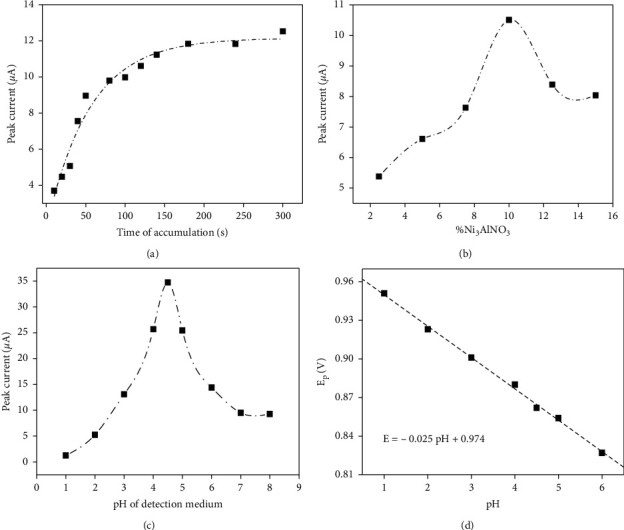
(a) Effect of accumulation time on the peak current of ISO; (b) effect of the amount of NiAl-LDH in the composition of the paste on the peak current of ISO; (c) effect of pH of detection medium on the peak current of ISO; (d) variation of the peak potential versus pH of the detection medium. Other conditions are the same as in Figure 4.

The effect of accumulation time is shown in Figure 6(a). The peak current increased gradually with the accumulation time up to 150 s. Afterwards, the peak current increased much slightly as with further increase in the accumulation time. This phenomenon could be attributed to the saturated adsorption of ISO on NiAl-LDH/CPE. Considering sensitivity, the optimal accumulation time of 150 s was employed in further experiments.

#### 3.4.2. The Effect of NiAl-LDH Amount on the Paste Composition

The study of the effect of NiAl-LDH proportion within the CPE was also expected to affect the electrode response.  Figure 6(b) presents the evolution of the SWV peak current when the detection of ISO was performed with NiAl-LDH/CPE prepared with various amounts of NiAl-LDH. The peak current was shown to increase with the amount of NiAl-LDH incorporated in the paste, up to a maximum value of 10%. This was followed by the decrease of the current for higher amount of LDH within the paste. From 2.5% to 10%, the increase on peak current was due to the number of adsorption sites, which increases at the solution electrode interface. The reduction of peak current observed at 12% is due to the fact that LDHs are weakly conductive material. The high amount of this modifier within the carbon paste reduced the conductivity of the electrode [33]. The optimum percentage of NiAl-LDH/CPE incorporated into the CPE was chosen to be 10% due to the best compromise between the number of sites and the conductivity of the paste.

#### 3.4.3. Influence of the pH of the Detection Medium

The acidity of the detection medium is a key parameter that can affect the mass transport to the electrode surface, especially when the redox process involves some protons as in the present case. The influence of the pH on the ISO response was studied at NiAl-LDH/CPE between pH 1 to 6, because ISO can be partially hydrolyzed in alkaline medium [32]. It is observed from Figure 6(c) that the peak current increased with an increase of pH of the detection medium to a maximum value at pH 4.5. After this value, the decrease of sensibility with increase of pH value was observed. For higher pH values, low current value was observed. The low electrode response obtained for pH < 2 may be due to prior protonation of amino group in the substrate, which induces electrostatic repulsion between the analyte and NiAl-LDH edges layer which contains positive charge. The sensibility of the electrode is optimal at pH 4.5. The lower stripping signal recorded for pH > 4.5 must probably be due to the quantity of OH^−^ in the medium which can react by exchange mechanism with exchanger ion in the interlayer, thus contributing to the reduction of the sensibility of the electrode.

The relationship between the oxidation peak potential and pH was shown in Figure 6(d). A linear shift of E_pa_ towards negative potential with an increasing pH indicated that protons are directly involved in the oxidation of ISO. It obeys the following equation: E_pa_(V) = −0.025pH + 0.974. The slope of the variations in *E*p vs. pH, of 0.025 V/ΔpH, suggests that the same numbers of protons and electrons are involved in the electrochemical oxidation of ISO, almost matching the theoretical Nernst equation [34].

#### 3.4.4. Influence of the SWV Technique Parameters

Square wave parameters are also important in controlling the peak intensity. These parameters include the frequency, the electrolysis potential, and amplitude. All these parameters were studied and the most suitable values used for the detection of ISO are given in Table 1.

#### 3.4.5. Interference Studies

Under optimal experimental conditions, the effects of other coexisting substances and anions were studied. The results summarized in Table 2 show that 5-fold excess of Cl^−^, NO_3_^−^, and HCO_3_^−^ did not interfere with the analysis of ISO. However, when the concentration of these ions increases up to 50-fold excess (Cl^−^, NO_3_^−^, and HCO_3_^−^), they interfere with the analysis of ISO by increasing the peak current due to the ion exchange properties of NiAl-LDH. We have also studied the effect of cations (Al^3+^, Pb^2+^). The results obtained show that Al^3+^and Pb^2+^ were found to interfere slightly at 50-fold excess with the analysis of ISO. The effect of anionic dye (orange II) shows that it interferes with ISO when the concentration is 10 times larger than ISO.

#### 3.4.6. Influence of ISO Concentration

**Figure 7:**
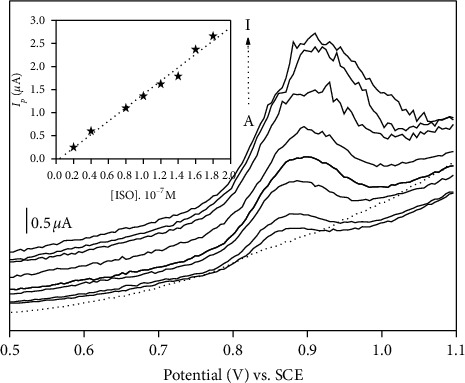
Dependence of the SWV peak current with increased ISO concentration from (a) to (i) 2 × 10^−8^; 4 × 10^−8^; 8 × 10^−8^; 1 × 10^−7^; 1.2 × 10^−7^; 1.4 × 10^−7^; 1.6 × 10^−7^; 1.8x10^−7^M. The inset shows the corresponding calibration curve.

The relationship between the oxidation peak current of ISO and the concentration was studied by SWV under optimized conditions (Figure 7). The peak current increases with the concentration of ISO over the range from 2 × 10^−8^ to 1.8 × 10^−7^ M. The relationship between these two parameters is linear, with a slope (*μ*A/M) of 0.14 and a correlation coefficient of 0.998. The detection limit for this work was estimated to be 1 × 10^−9^ mol L^−1^ on the basis of signal-to-noise ratio equal to 3. A comparison of the performance of NiAl-LDH/CPE including the limit of detection and the linear range with those reported in the literature is shown in Table 3 which indicates that the proposed sensor exhibited detection limits lower than those reported by certain authors.

The analytical applicability of the modified electrode was applied to the determination of ISO in real sample. A volume of 50 mL of the spring water was first analyzed by using the optimized parameters established in this study and ISO was not detected. However, if these samples were spiked with 0.16 *μ*mol L^−1^ of ISO, the content was determined by the standard addition method (the results are summarized in Table 4). The obtained values are in good agreement with the spiked value, indicating that the proposed method is a good alternative for the analytical determination of this pesticide in the sample.

## 4. Conclusion

In this work, NiAl-LDH was shown to be effective material for the elaboration of an amperometric sensor for ISO. Prior to its use for sensing purposes, this material was characterized by X-ray diffraction, infrared spectroscopy, and thermal analysis. The determination of ISO was examined at a bare CPE and NiAl-modified CPE by using square wave voltammetric techniques. Results showed that the peak current was greatly enhanced (more that 2.6-fold) compared to the response obtained using bare CPE. It was also found that the sensitivity of this modified electrode depends on the loading of NiAl-LDH on the paste composition and mainly on the parameters involved in the detection step by square wave voltammetry. After optimization, a detection limit of 1x10-9 mol.L^−1^ was achieved. The proposed method was applied to quantify ISO in real media. The obtained results clearly indicated that the proposed voltammetric procedure could be applied for ISO sensing in environmental polluted media.

## Figures and Tables

**Table 1:** Optimum experimental conditions used in SWV of ISO.

Instrumental parameter	Range examined	Optimum value
Electrolysis potential (mV)	−0.2 to 1.6	0.8
Frequency (Hz)	25 to 220	125
Amplitude (V)	25 to 100	50

**Table 2:** Effect of interference ions on the response of the NiAl-LDH/CPE to 25 *μ*M ISO in acetate buffer (pH 4.5).

Interference ions	Added amount over [ISO]	% variation in the anodic peak current (Ip = 100%)
Cl^−^	5	0
	25	+32.1
	50	+52.4

NO_3_^−^	5	0
	25	+21.7
	50	+33.21

Orange II	10	+10.9
	25	+21.4
	50	+54.45

HCO_3_^−^	50	+33.26

Al^3+^	50	−1.5

Pb^2+^	50	−3

**Table 3:** Comparison of the proposed method with literature methods for the determination of phenyl urea herbicides.

Electrode configuration	Linearity range (M)	Detection limit (M)	Ref
Graphene modified/^*∗*^GCE	9.69 × 10^−8^ -4.84 × 10^−5^	9.69 × 10^−8^	[10]
GO-MWCNT film-modified/^*∗*^GCE	9 × 10^−6^–0.38 × 10^−3^	0.645 × 10^−6^	[31]
Polypyrrole/^*∗*^GCE	2.42.10^−9^–1.42.10^−6^	2.42 × 10^−9^	[32]
Sodium montmorillonite film/^*∗*^GCE	0.206 × 10^−6^–61.8 × 10^−6^	0.206 × 10^−6^	[35]
Organomontmorillonite/^*∗*^GCE	4.84 × 10^−9^–1.45 × 10^−6^	4.84 × 10^−9^	[36]
PANI/MWCNTs/^*∗*^GCE	4.84 × 10^−8^–4.84 × 10^−4^	4.84 × 10^−10^	[37]
Well-jet electrode	4.84 × 10^−7^–7.27 × 10^−3^	4.84 × 10^−7^	[38]
Ph-CN-SWCNT/^*∗*^GCE	1 × 10^−6^–2 × 10^−4^	0.20 × 10^−6^	[39]
NiAl-LDH/^*∗∗*^CPE	2 × 10^−8^–1.8 × 10^−7^	1 × 10^−9^	This work

^*∗*^GCE: glassy carbon electrode. ^*∗∗*^CPE: carbon paste electrode. GO-MWCNTs: graphene-multiwalled carbon nanotubes. PANI/MWCNTs: polyaniline multiwalled carbon nanotubes. Ph-CN-SWCNT: phthalocyanine-single walled carbon nanotube.

**Table 4:** Determination of ISO in spring water.

	ISO added (M)	ISO found (M)	Recovery (%)
Spring water	0.160(*μ*M)	0.156 ± 0.012 *μ*M	97.5

^a^Number of samples assayed = 5.

## Data Availability

The data used to support the findings of this study are available from the corresponding author upon request.

## Supplementary Materials

Supplementary Materials Linear relation of anodic peak current against the square root of scan rate of 10^−4^ M of [Fe(CN)_6_]^3−^ in KCl 0.1 M: (a) bare CPE and (b) NiAl-LDH/CPE. Insets were the CVs of bare EPC (a) and NiAl**−**LDH/CPE (b) in 10^−4^ M [Fe(CN)_6_]^3−^ at different scan rates from 10 to 80 mVs^−1^.
