# Supplementary material for: A Low-Cost Layered Double Hydroxide (LDH) Based Amperometric Sensor for the Detection of Isoproturon in Water Using Carbon Paste Modified Electrode
Source: J Anal Methods Chem. 2020 Aug 20;2020:8068137. doi: 10.1155/2020/8068137 (PMC7480358; doi:10.1155/2020/8068137)
Supplement: Supplementary Materials — Linear relation of anodic peak current against the square root of scan rate of 10−4 M of [Fe(CN)6]3− in KCl 0.1 M: (a) bare CPE and (b) NiAl-LDH/CPE. Insets were the CVs of bare EPC (a) and NiAl−LDH/CPE (b) in 10−4 M [Fe(CN)6]3− at different scan rates from 10 to 80 mVs−1. [file 8068137.f1.docx]

**Supplementary Material**

**A Low-Cost Layered Double Hydroxide (LDH) based amperometric sensor for the detection of isoproturon in water using carbon paste modified electrode**

**H. L. Tcheumi^*1,2^ , A .P. Kameni Wendji^1^, I. K. Tonle,^1,3^ E. Ngameni^1^**

(1) Laboratoire de Chimie Analytique, Département de Chimie Inorganique, Faculté de Sciences, Université de Yaoundé I. BP 812 Yaoundé, Cameroun.

(2) Laboratoire de Chimie de l’Environnement, Département des Sciences Environnementales, Ecole Nationale Supérieure Polytechnique de Maroua, Université de Maroua BP 46 Maroua, Cameroun.

(3) Laboratoire de Chimie Minérale, Département de Chimie, Faculté des Sciences, Université de Dschang. BP 67 Dschang, Cameroun.

**The electroactive surface area of CPE and NiAl-LDH/CPE**

In order to illustrate that Layered Double Hydroxide material used as electrode modified could improve the surface area, electroactive surface area (A) of bare CPE and NiAl-LDH/CPE was determined by Cyclic voltammogram (CV) in 10^-4^ M of [Fe(CN)_6_]^3-^ in KCl 0.1 M solution at different sweep rates (v) according to the Randles-Sevcik equation [1]:

As shown in Fig. S1a and Fig. S1b, both the anodic peak currents (I_pa_) of CPE and NiAl-LDH/CPE were proportional to the square root of scan rate. Therefore, with the constant parameters of D_o_, C_o_ and n, we could successfully obtain an approximate value of A according to the Randles-Sevcik equation. The electroactive surface area of 0.047 cm^2^ and 0.055 cm^2^ for CPE and NiAl-LDH/CPE were acquired, respectively. The electroactive surface area of NiAl LDH/CPE is 1.17 time that of bare CPE.

**Figure S1 :** Linear relation of anodic peak current against the square root of scan rate of 10^-4^ M of [Fe(CN)_6_]^3-^ in KCl 0.1 M. (a) : bare CPE and (b) : NiAl**-**LDH/CPE. Insets were the CVs of bare EPC (a) and NiAl**-**LDH/CPE (b) in 10^-4^ M [Fe(CN)_6_]^3-^ at different scan rates from 10 to 80 mVs^-1^

**References**

1. A. J. Bard, L. R. Faulkner. Electrochemical methods: fundamentals and applications, 2nd ed., Wiley, New York, 1980.
